# Pooled Sequencing of 531 Genes in Inflammatory Bowel Disease Identifies an Associated Rare Variant in *BTNL2* and Implicates Other Immune Related Genes

**DOI:** 10.1371/journal.pgen.1004955

**Published:** 2015-02-11

**Authors:** Natalie J. Prescott, Benjamin Lehne, Kristina Stone, James C. Lee, Kirstin Taylor, Jo Knight, Efterpi Papouli, Muddassar M. Mirza, Michael A. Simpson, Sarah L. Spain, Grace Lu, Franca Fraternali, Suzannah J. Bumpstead, Emma Gray, Ariella Amar, Hannah Bye, Peter Green, Guy Chung-Faye, Bu’Hussain Hayee, Richard Pollok, Jack Satsangi, Miles Parkes, Jeffrey C. Barrett, John C. Mansfield, Jeremy Sanderson, Cathryn M. Lewis, Michael E. Weale, Thomas Schlitt, Christopher G. Mathew, UK IBD Genetics Consortium

**Affiliations:** 1 Department of Medical and Molecular Genetics, Kings College London, Guy’s Hospital, London, United Kingdom; 2 Department of Epidemiology and Biostatistics, Imperial College London, London, United Kingdom; 3 Department of Medicine, University of Cambridge School of Clinical Medicine, Cambridge Biomedical Campus, Cambridge, United Kingdom; 4 Campbell Family Mental Health Research Institute, Centre for Addiction and Mental Health, Toronto, Ontario, Canada; 5 Department of Psychiatry, University of Toronto, Toronto, Ontario, Canada; 6 NIHR GSTFT/KCL Comprehensive Biomedical Research Centre Genomics Core Facility, King’s College London School of Medicine, Guy’s Hospital, London, United Kingdom; 7 Randall Division of Cell and Molecular Biophysics, King’s College London, Guy’s Campus, London, United Kingdom; 8 Wellcome Trust Sanger Institute, Wellcome Trust Genome Campus, Hinxton, Cambridge, United Kingdom; 9 Department of Gastroenterology, King’s College Hospital NHS Foundation Trust, Denmark Hill, London, United Kingdom; 10 Gastroenterology and Hepatology, St George’s Healthcare NHS Trust, Tooting, London, United Kingdom; 11 Gastrointestinal Unit, Molecular Medicine Centre, University of Edinburgh, Western General Hospital, Edinburgh, United Kingdom; 12 Institute of Genetic Medicine, Newcastle University, Newcastle upon Tyne, United Kingdom; 13 Guy’s & St Thomas’ NHS Foundation Trust, St Thomas’ Hospital, Department of Gastroenterology, London, United Kingdom; 14 Division of Medical Biochemistry, University of Cape Town, Cape Town, South Africa; University of Oxford, UNITED KINGDOM

## Abstract

The contribution of rare coding sequence variants to genetic susceptibility in complex disorders is an important but unresolved question. Most studies thus far have investigated a limited number of genes from regions which contain common disease associated variants. Here we investigate this in inflammatory bowel disease by sequencing the exons and proximal promoters of 531 genes selected from both genome-wide association studies and pathway analysis in pooled DNA panels from 474 cases of Crohn’s disease and 480 controls. 80 variants with evidence of association in the sequencing experiment or with potential functional significance were selected for follow up genotyping in 6,507 IBD cases and 3,064 population controls. The top 5 disease associated variants were genotyped in an extension panel of 3,662 IBD cases and 3,639 controls, and tested for association in a combined analysis of 10,147 IBD cases and 7,008 controls. A rare coding variant p.G454C in the *BTNL2* gene within the major histocompatibility complex was significantly associated with increased risk for IBD (p = 9.65x10^−10^, OR = 2.3[95% CI = 1.75–3.04]), but was independent of the known common associated CD and UC variants at this locus. Rare (<1%) and low frequency (1–5%) variants in 3 additional genes showed suggestive association (p<0.005) with either an increased risk (*ARIH2* c.338-6C>T) or decreased risk (*IL12B* p.V298F, and *NICN* p.H191R) of IBD. These results provide additional insights into the involvement of the inhibition of T cell activation in the development of both sub-phenotypes of inflammatory bowel disease. We suggest that although rare coding variants may make a modest overall contribution to complex disease susceptibility, they can inform our understanding of the molecular pathways that contribute to pathogenesis.

## Author Summary

Crohn’s disease and ulcerative colitis are two forms of inflammatory bowel disease which cause chronic inflammation of the gastrointestinal tract. Common genetic variants in more than 160 regions of the human genome have been associated with an altered risk of these disorders, but leave much of the estimated genetic contribution to disease risk unexplained. We sought to establish whether rare genetic variants which alter the structure or function of the proteins encoded by genes also contribute to disease susceptibility. We used high throughput DNA sequencing to screen over 500 genes for such variants in nearly 500 patients and controls, and validated interesting variants in about 10,000 patients and 7,000 controls. We detected association of a limited number of rare variants from coding regions with disease, suggesting that they do not account for a large proportion of genetic susceptibility. However, they highlight the involvement of genes of potential importance in the development of inflammatory bowel disease, including those involved in the activation of immune cells, the regulation of immune response genes, and the degradation of proteins in cells.

## Introduction

The inflammatory bowel diseases (IBD), Crohn’s disease (CD) and ulcerative colitis (UC) are chronic inflammatory disorders of the gastrointestinal tract that can cause diarrhoea, abdominal pain, bleeding and weight loss. Collectively they affect approximately 827 per 100,000 individuals in European populations and their incidence is rising [1]. CD may affect any part of the gut with discontinuous penetrating lesions, whereas in UC the disease is limited to the colon and rectum and the lesions are continuous but superficial [2]. Both diseases are multi-factorial, with a complex aetiology that involves a combination of an underlying genetic predisposition and environmental triggers. A variety of factors have been proposed to contribute to the pathogenesis including changes within the intestinal microbiota, a defective mucosal barrier, and / or dysregulation of the immune response [3].

A meta-analysis of genome-wide association studies (GWAS) in CD and UC by the International IBD Genetics Consortium (IIBDGC), followed by extensive confirmation of association signals in more than 75,000 individuals has increased the number of IBD-associated loci to 163 [4]. The majority of these loci are associated with both CD and UC, which suggests that there is extensive overlap in the biological mechanisms involved in their pathogenesis. However, although our understanding of the aetiology of IBD has been substantially advanced by GWAS-based approaches, only a modest proportion of total disease variance can be explained by current genetic findings (<15%) [4]. It has been proposed that rare coding sequence variants may make a substantial contribution to disease variance, and confer disease risks large enough to warrant use in preventative screening [5]. Such variants would not be detectable by a conventional GWAS approach because they are not well tagged by the common SNPs on which GWAS panels are based [6].

New high throughput DNA sequencing technologies have made it feasible to investigate the contribution of rare variants to complex disease. In CD, it has long been known that low frequency coding variants in *NOD2* make a substantial contribution to disease risk [7–9], and more recent high-throughput sequencing strategies have discovered several independent IBD associated rare variants in *NOD2* and other genes from GWAS loci including *IL23R*, *CARD9*, *IL18RAP*, *CUL2*, *C1orf106*, *PTPN22*, *RNF186* and *MUC19* [10–12]. However, a recent large-scale sequencing study of the coding regions of 25 autoimmune candidate genes in more than 40,000 individuals yielded little evidence that rare variants drive the associations observed at susceptibility loci for common immune disorders, including CD [13]. Thus the exact contribution of rare coding variants to IBD and other immune disorders remains unknown.

Here we describe a targeted high throughput sequencing approach in pooled DNA samples from 474 CD patients and 480 population controls to screen all exons, splice sites, and proximal promoter regions in 531 positional and functional candidate genes. We sequenced CD patients with early-onset disease and/or strong family history to enrich for functional causal variants with stronger effects, and we looked beyond common loci using functionally-derived bioinformatics data such as pathway and protein network analysis to identify additional candidate genes involved in key processes such as the immune-response and autophagy. Potential functional variants and those with evidence of association with CD underwent validation genotyping in a follow up study including 6507 IBD cases and 3064 controls with replication of the top hits in an additional 3662 IBD cases and 3639 controls giving a total of over 10,000 IBD cases and over 7,000 controls for the final combined analysis. We discovered significant novel association of a rare coding variant in *BTNL2* and suggestive associations of additional variants in potentially novel IBD genes.

## Results

### Sequencing of CD cases and controls

**Figure 1:**
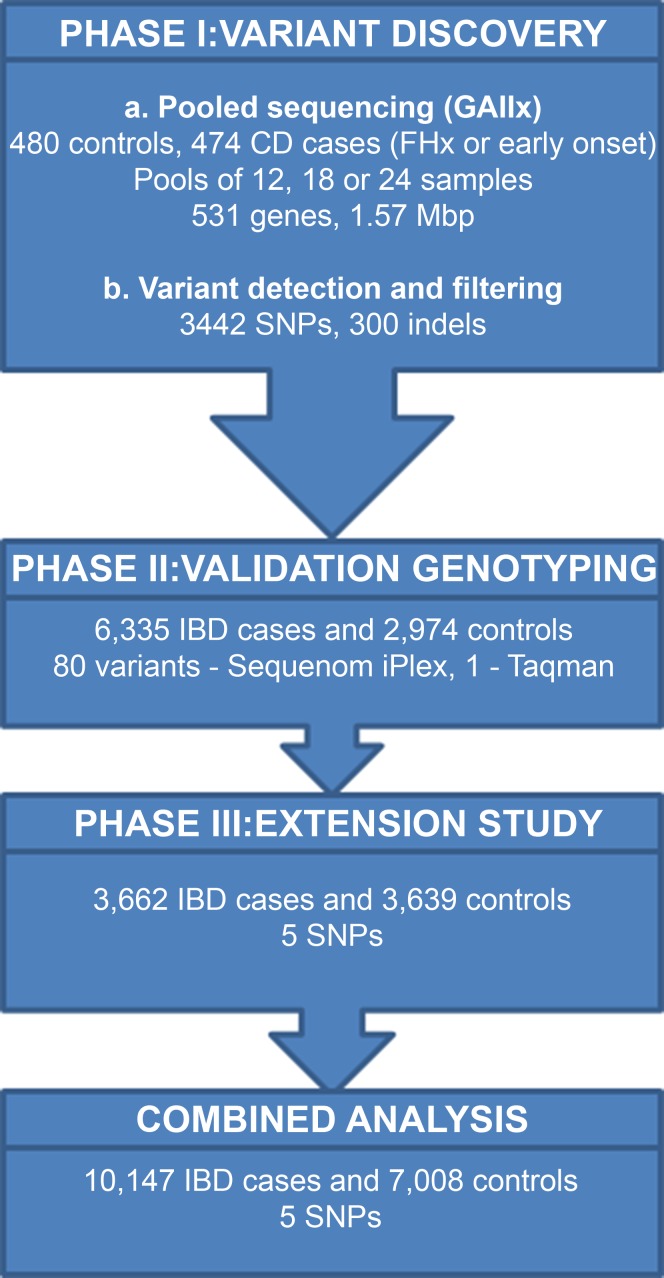
Summary of strategy for detecting rare variants associated with IBD. Overview of our rare variant screening strategy in IBD using DNA pools. We detected 3442 high quality variants in phase I based on stringent filtering criteria. We were able to validate 1252 of these variants using a) previously generated genotyping data for 153 SNPs in 634 of the individuals who were sequenced; b) case-control association p values for 1099 SNPs from CD Immunochip study [4]. We then performed validation genotyping of 80 variants in phase II in 6335 IBD cases and 2974 controls and extended the analysis of the top 5 SNVs to a further 3662 IBD cases and 3639 controls (phase III) to allow a final combined analysis of 10,147 IBD cases and 7,008 controls.

An overview of our strategy for the discovery of rare variants associated with CD is shown in Fig. 1. We selected 531 candidate genes for sequencing in phase I based on 5 selection criteria (Table 1 and described in Materials and Methods). A total of 6,249 exons, together with associated splice sites and proximal promoter regions, were sequenced in 474 CD cases and 480 population controls. Samples were sequenced in case-only or control-only pools of 12, 18 or 24 individuals using the Illumina Genome Analyzer II platform. An average of 98 million sequence reads were generated per pool, of which 87% could be aligned to the reference genome and 64% passed subsequent quality control steps (Materials and Methods). Of these, an average of 25.7 million reads mapped to the targeted genomic regions, which corresponded to a capture efficiency of 40.5%. We observed a mean read depth of >1000x per pool across the 1.57 million bases captured. Taking into consideration the number of individuals per pool, on average 90% of all bases had coverage greater than 4x per haploid genome (S1 Fig.).

In order to reduce false positives calls due to sequencing errors, we applied a stringent filtering procedure (Materials and Methods), after which the number of variants was approximately constant across all pools for all types of variants (S2 Fig.).

**Table 1:** Candidate gene selection strategies.

Gene selection criteria	Count
Genes in Crohn’s disease GWAS hit regions	75
Genes in GWAS hits from other auto-immune diseases	50
Genes identified by pathway analysis	74
Genes identified by literature search	214
Genes identified by network analyses	300
Total Genes on array	531
Total exons	6290
Total base pairs	1,569,003

Selection criteria are non-exclusive so that many genes were selected by more than one criterion.

**Figure 2:**
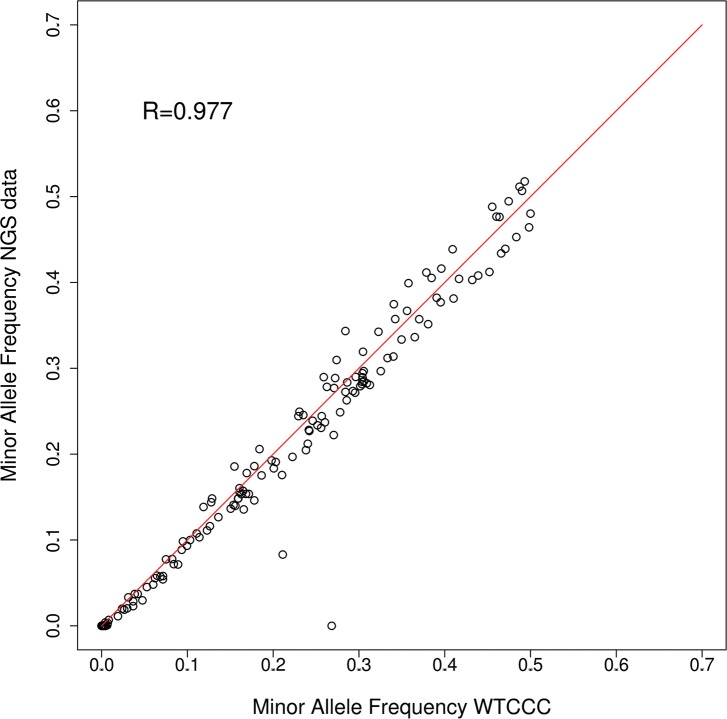
Minor allele frequencies WTCCC vs pooled NGS (24 pools combined). MAFs for 153 SNPs were compared between allele frequency estimates based on pooled NGS and genotyping data from the WTCCC [47] for 634 individuals. MAFs are strongly correlated between both datasets (Spearman rank correlation coefficient R = 0.976), with only two SNPs showing substantial differences.

Next, variant allele frequencies in each pool were estimated from base-call counts. We assessed the accuracy of this approach by comparing these estimates to minor allele frequencies (MAFs) derived from genotyping data generated by the Wellcome Trust Case Control Consortium (WTCCC); genotypes were available for 153 SNPs located in the captured genomic regions in 66.5% (388 controls, 246 cases) of the individuals sequenced in this study [14]. We observed a very strong correlation (Spearman Rank Correlation r = 0.977) between MAFs for the WTCCC genotypes and the pooled sequencing data (Fig. 2).

After filtering, 3,749 single nucleotide variants (SNVs, here used to refer to any single nucleotide variation regardless of minor allele frequency) were retained, of which over half were low frequency (<5%, S1 Table). Just over half of the SNVs were located in exons (51.1%; 1914 SNVs), with the remainder located in introns, untranslated regions (UTRs), putative splice sites and intergenic regions. We considered 106 of the SNVs (3%) to be novel because they were not present in dbSNP138 (http://www.ncbi.nlm.nih.gov/SNP/). Analysis of all SNVs yielded a transition/transversion ratio (Ti/Tv) of 2.41, which is expected given the bias toward coding sequences in our target regions and is in agreement with previous studies [11]. In addition to SNVs we identified 183 deletions and 117 insertions. Only 14 of these insertion/deletions (indels) were located in an exon (S1 Table). A high rate of true positives in our sequencing data was corroborated by the presence of 97% of our variants in dbSNP138, and the strong correlation between MAFs for the pooled sequencing data and the WTCCC genotype data. Regarding sensitivity of variant detection, the regions captured in our sequencing contain 1,599 variants with a MAF >5% in the phase I release of the 1000 Genomes project, 1,291 of which (80.7%) were detected in our pooled sequencing data.

### Analysis of bias

**Figure 3:**
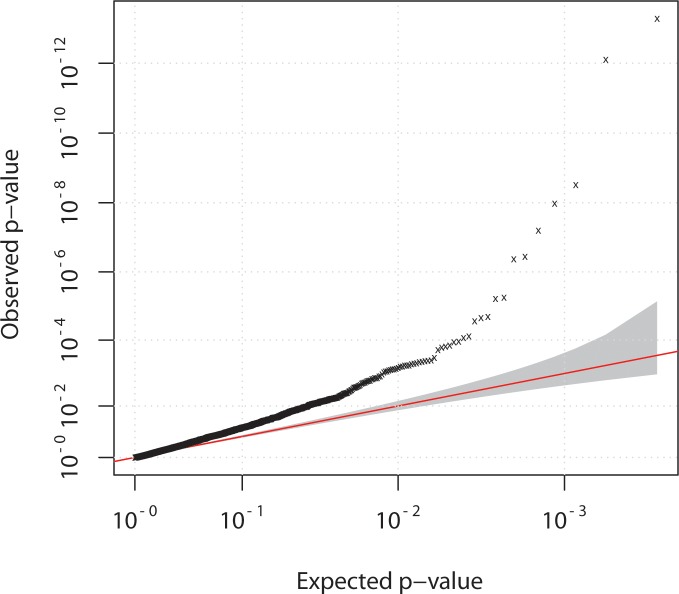
Quantile-quantile plot of chi-squared statistic. Data for case-control comparison of allele frequencies of 3442 variants, detected in pooled sequencing experiment for 42 case and 40 control pools. Overall genomic inflation (lambda) of 1.3 was observed.A genomic control correction was therefore applied for downstream association analysis (Materials and Methods).

Our strategy relied on the necessity of sequencing individuals in case-only or control-only DNA pools which could potentially inflate any biases that would arise due to sequencing batch effects. We therefore used principal component analysis to control for this and identify any outlier pools. Examination of PC axes 1 and 2 revealed pools 7 and 8 to be outliers. Both were case pools, although each represented a single lane of flow-cell data from two different runs of the GAII sequencer. Once these pools were removed the data showed reasonable separation of points, but there was a clear tendency for case and control pools to be separated along PC axis 1 (S3 Fig.), which led to an overall genomic inflation of 1.3 (Fig. 3). The extent of the systematic bias in the data meant that PC axes could not be used as covariates in a logistic regression to correct for it, as previously noted [15], nor could we apply methods designed to correct for overdispersion but not bias [16]. We therefore applied a genomic control method for downstream association analysis plus additional QC measure for removal of SNVs with strong over dispersion among pools (Materials and Methods). We note that it is possible that the high systematic bias reflects genuine causal influences given the candidature of all the genes sequenced, but equally we cannot exclude the possibility of experimental sources of bias.

### Association analysis

Variant level association with case-control status of pools was performed using logistic regression on 3,442 SNVs after exclusion of 307 SNVs that were too rare (had zero count in case or controls), or only had allele counts in excluded pools (Materials and Methods) (Fig. 3). Encouragingly, several known common and low frequency CD susceptibility variants were detected including variants in *ATG16L1*, *IRGM*, *IL23R*, *CARD9* and *NOD2*, and rare variants in *IL23R* and *NOD2* [7, 8, 10, 11, 17], all of which showed the expected CD odds ratios and allele frequencies in both cases and controls (S2 Table).

We noticed that 1,099 of 3,442 SNVs tested for association in our sequencing data were either included in the IBD Immunochip project directly (803) or by a suitable tagging SNP (r^2^≥0.8, n = 296) [4]. The IBD Immunochip dataset was therefore considered as an independent replication study for these 1099 variants. We found that 43 of the 141 variants (30.5%) that were at least nominally associated in our sequencing data (p<0.05) were also associated in the CD Immunochip data (p<5x10^−8^), resulting in a significant correlation between the two datasets (r = 0.446, p = 4.46x10^−32^).

The majority of variants identified by our study were rare, resulting in modest statistical power for the SNV-wise tests of association. We therefore applied gene-level association tests to investigate whether the burden of predicted functional variants non-synonymous and stop-gain variants) was different in cases compared to controls (**Materials and Methods**). In our discovery sequencing we identified 341 genes containing one or more functional variants. Thus the gene-burden test provided >90% power to detect a gene-level association where the cumulative MAF is 5% and the cumulative risk (OR) is 2.5 at an alpha level of 0.00015 (allowing for Bonferroni correction based on 341 genes/tests). We identified significant gene-level associations for *BTNL2* (no. of variants = 18, p = 8.15x10^−5^) and *NOD2* (no. of variants = 10, p = 9.03x10^−6^) (S3 Table). Since both genes contained substantially more functional variants than other genes that were tested we controlled for LD by permutation analysis (Materials and Methods), which resulted in loss of significance for *BTNL2* (p = 0.022), whilst *NOD2* remained significant (p<0.001). Repeating the analysis to include all intragenic variants (functional and non-functional) gave a similar outcome, although neither gene survived permutation testing (p>0.001).

### Validation genotyping and extension study

In Phase II we selected 85 variants for validation of disease association by Sequenom (84 SNVs) or Taqman (1 SNV) genotyping in 6,335 IBD cases from the UK IBD Genetics Consortium (3,715 CD and 2,619 UC) and 2,974 controls (Materials and Methods). UC cases were included in the validation because of the extensive overlap in known associated loci for these two related phenotypes [4]. SNVs were selected based on at least nominal evidence of association in the pooled sequencing experiment (p < 0.05), and we prioritised those predicted to be functionally relevant (S1 Text). SNVs already genotyped as part of the IBD Immunochip experiment [4] were excluded. Post-genotyping quality control revealed that two SNVs failed to genotype, two were non-polymorphic and one was not in Hardy Weinberg equilibrium (p < 1x10^−6^ in controls) leaving a total of 80 SNVs (S4 Table). The genotyping call rate for all remaining SNVs was >90%. To allow validation of our variant calling analysis pipeline we genotyped an additional subset of 368 individuals previously included in our sequencing experiment and were able to show strong correlation between predicted and actual allele frequencies for all 80 SNVs (r = 0.94, p = 2.42x10^−38^) and low frequency SNVs (MAF<5%, r = 0.86, p = 1.69x10^−24^). In addition, allele frequencies derived from the pooled sequencing experiment were compared to those derived from all individuals in the phase II genotype data and revealed a highly significant correlation (r = 0.971, p < 6.58x10^−48^), further supporting the validity of the pooled sequencing approach. We followed up 3 insertion deletion polymorphisms by Taqman genotyping in 2,532 IBD cases and 3,545 controls (rs58682836/*COBL* frameshift delTTC, rs71297581/*TYK2* upstream insC, and rs3833864/*PIK3C* upstream insC). The indel rs71297581 failed genotyping quality control, producing poor genotype clusters, and neither rs58682836 nor rs3833864 were associated with IBD (p > 0.5).

There was some evidence of association (p < 0.05) for 16 SNVs across 12 genes, *CHTOP*, A*RIH2*, *NICN1*, *PLSCR1*, *IL12B*, *BTNL2*, *QRSL1*, *CALML5*, *GLT1D1*, *RTEL1*, *ATG4B* and *TBX21* (S5 Table). These were associated with either CD (11 variants), UC (5 variants) or IBD (12 variants), with 4 of these variants located in *BTNL2*. *BTNL2* and *IL12B* map to established UC and IBD risk loci and have previously been implicated in UC and IBD respectively (6p21/HLA class II/UC and 5q31/IBD respectively), whilst *ARIH2* and *NICN1*are within the same previously described IBD locus (3p21.3/IBD) but the genes themselves have not been implicated. Association of the other 10 genes and their respective variants with IBD has not been reported previously.

Since *BTNL2* is within the MHC region and close to the common IBD associated locus in the HLA class 2 region we investigated the extent of LD across the 4 variants and their independence from the known risk locus using haplotype and conditional analysis within a set of cases and controls previously genotyped in both the Immunochip study and our follow up genotyping study (**Materials and Methods**). The analysis showed that the rare *BTNL2* variants p.G454C and p.D336N (rs28362675 and rs41441651) were in almost complete LD with each other (r^2^ = 0.99) and remained associated with IBD even when the effect at the common SNPs was accounted for (p < 0.049), as did *BTNL2* c.-118G>T (rs28362684, p = 0.039) but not the missense variant (p.S334L). Regarding association of the 80 variants with IBD, only the two highly correlated variants in *BTNL2* (p.454C and p.D336N) surpassed the Bonferroni threshold for multiple testing (p < 0.0006 for 79 independent SNVs tested). However there was significant enrichment for association signals among the 79 variants, with nearly 3 times the number of significant results than would be expected by chance, with 14% of p-values for association with IBD (i.e. 11/79) being less than 0.05 (p = 0.00189).

Recognising the relatively low power of the validation panel to detect significant association of rare variants with disease, we next carried out extended genotyping (Phase III) of the 5 top SNVs that had a p < 0.01 (and in the case of *BTNL2* were independent of each other and the known common risk variants) in an additional panel of 3,662 IBD cases and 3,639 controls (Materials and Methods), and then performed a combined case-control analysis of all 10,147 IBD cases and 7,008 controls that were either sequenced or genotyped (Table 2). We confirmed a genome-wide significant association with *BTNL2* p.G454C and increased risk of IBD at (p = 9.65x10^−10^, OR = 2.3 [95%CI = 1.75–3.04]). We detected association for 3 other variants of the 5 tested in phase III (p < 0.005). Notably, in the combined analysis the direction of the effect for each of the 5 SNPs is consistent with the effect in the validation panel (p < 0.031). However, the 3 additional associations do not meet correction for 79 independent tests (P<0.00063) and are therefore suggestive. They include two low frequency missense variants *IL12B* p.V298F and *NICN1* p.H191R associated with a reduced risk for IBD and one noncoding variant *ARIH2* c.338-6C>T which was associated with an increased risk (Table 2). Two of the 3 missense variants associated with IBD (*IL12B* p.V298F and *BTNL2* p.G454C) were predicted to be damaging or non-tolerated by Polyphen2 [18] and/or SIFT (sorts intolerant from tolerant) or Provean [19]. *IL1B* encodes the p40 subunit common to both the interleukin-12 and interleukin-23 heterodimeric cytokines. The p.V298F variant is not in LD with the common risk variant at this locus (r^2^ = 0.001, D’ = 0.079), and is predicted to disrupt the structure of the p40 protein by the mCSM structure prediction tool [20], with a predicted stability change ΔΔG of −0.917. We also used the available structure of the IL12B (p40) and IL23A (p19) proteins to model the effect of the V298F mutation in *IL12B* (S4 Fig.). This indicated an altered conformational state of a region of p40 which is important for binding to its partner proteins IL23A (p19) and IL12A (p35) [21].

**Table 2:** Combined case-control association analysis of 5 sequence variants from the phase III extension study in 10,147 IBD cases and 7,008 controls from phases I–III.

Gene, variant	dbSNP	Chr:bp	Control	Crohn’s disease			Ulcerative colitis			Inflammatory bowel disease		
			MAF	MAF	P	OR (95%CI)	MAF	P	OR (95%CI)	MAF	P	OR (95%CI)
*ARIH2*	rs200140527	3:49004552	0.09%	0.22%	0.01086	2.34	0.22%	0.01504	2.32	0.22%	0.004407	2.39
c.338-6C>T						(1.19–4.61)			(1.15–4.67)			(1.29–4.42)
*NICN1*	rs61729946	3:49462458	1.30%	0.90%	0.003094	0.69	0.99%	0.03548	0.76	0.94%	0.001868	0.72
p.H191R						(0.54–0.88)			(0.59–0.98)			(0.59–0.89)
*IL12B*	rs3213119	5:158743788	3.24%	2.54%	0.001242	0.78	2.80%	0.06153	0.86	2.66%	0.001825	0.82
p.V298F						(0.67–0.91)			(0.74–1.01)			(0.72–0.93)
*BTNL2*	rs28362675	6:32362521	0.47%	0.90%	3.63x10^−05^	1.91	1.31%	3.48x10^−12^	2.79	1.08%	9.65x10^−10^	2.31
p.G454C						(1.40–2.61)			(2.06–3.76)			(1.75–3.04)
*TBX21*	rs41444548	17:45811354	7.70%	7.19%	0.1279	0.93	6.97%	0.03644	0.9	7.09%	0.03358	0.91
c.491+43C>G						(0.84–1.02)			(0.81–0.99)			(0.84–0.99)

### *BTNL2*

*BTNL2* is located on chromosome 6p21.3, which contains two common and independent risk loci for IBD. The closest (approximately 200Kb proximal to *BTNL2*) is within the HLA class II region and is associated with UC (rs477515, p = 5x10^−133^). The other locus is much further away (approximately 1.1Mb distal of *BTNL2*) within the HLA-class I region, and associated with CD (rs9264942, p = 5x10^−28^) [4]. We observed that *BTNL2* p.G454C was associated very strongly with UC (p = 3.5x10^−12^, Table 2) and also associated with CD but to a lesser extent (p = 3.6x10^−5^, Table 2). In view of the extended LD in this region, it is possible that these associations could be due to LD with the known common risk variants in the HLA class I or class II regions. We investigated this by further conditional logistic regression analysis using 1,638 IBD cases and 1,243 controls genotyped in both the Immunochip study and both our genotyping studies. We confirmed that *BTNL2* p.G454C was not in LD with either of the two common IBD risk variants (r^2^ < 0.001, D’ < 0.7). Conditional analysis showed that *BTNL2* p.G454C remained significantly associated with IBD when the effect at the common UC associated SNP (rs477515) was accounted for (p = 0.0045, S6 Table), or the common CD associated SNP (rs9264942) was accounted for (p = 4.83x10^−5^, S6 Table). Haplotype analysis showed that the risk “A” allele for the rare variant occurred on haplotypes containing either the non-risk or the risk allele for both of the common variants, further suggesting their independence. Given the strength of the effect of p.G454C in UC individuals in particular (Table 2) we carried out specific haplotype analysis using this and the common UC GWAS SNP in the class II HLA region and showed that haplotype **A-A** containing the risk allele at the rare variant (p.G454C) and the non-risk “A” allele at the common UC GWAS SNP (rs477515) respectively, although very rare, was increased in frequency in cases, (0.2%) compared to controls (0.07%) (S7 Table), and the haplotype G-A containing the risk allele at both the common and the rare variant had a much higher risk for disease (OR = 6.51 [95%CI = 1.87–22.72]) than the haplotype G-C that only had the risk allele at the common SNP and lacked the rare risk allele (OR = 1.38 [95%CI = 1.20–1.57]).

## Discussion

In this study we investigated the contribution of rare variants to susceptibility to inflammatory bowel disease in a large set of candidate genes. Use of targeted next generation sequencing in combination with a DNA pooling strategy allowed us to screen over 500 genes for variants in more than 900 individuals, which is ten-fold more than were investigated in previous studies of IBD [10–12]. The results demonstrate that this is a cost-effective strategy for identifying low frequency variants that may be associated with disease. We were able to validate our approach by accurate estimation of the minor allele frequencies of 153 SNPs previously genotyped in individual case and control samples by the Affymetrix 500K SNP array, and by successfully reproducing the effect sizes (odds ratios) and allele frequencies of multiple common and low-frequency variants previously associated with IBD. We also demonstrated highly significant overlap of association for 1,099 SNPs that were common to our study and the recent GWAS/Immunochip meta-analysis for IBD [4], and showed a strong correlation between the allele frequencies and odds ratios of 80 SNVs that were genotyped by both pooled DNA sequencing and genotyping in our follow up study. Strong correlations between allele frequency estimates from pooled sequencing and genotyping have also been reported in previous studies of Crohn’s disease [10, 11], although read counts tended to underestimate actual frequencies for rare variants in one study [10]. However this approach could prove useful when supported by stringent quality control and validation measures.

Sequencing of coding and potential regulatory regions of 531 genes in a discovery set of 954 individuals, followed by genotyping in 17,131 individuals has allowed us to identify a novel disease associated genetic variant within a gene that maps to a region previously associated with IBD, and suggestive associations of other variants in a known IBD susceptibility gene and in other genes not previously implicated in IBD. The association of the rare variant p.G454C in *BTNL2* reached genome-wide significance, and was independent of the known common risk variants for IBD in the HLA region in both a conditional and haplotype analysis. However, this is a complex region of the genome with extensive allelic variation and linkage disequilibrium, and additional as yet unknown IBD risk variants at this locus may exist that are independent of the two main HLA signals previously described but correlated with our rare variant. The glycine residue is highly conserved across all mammals and the cysteine substitution is predicted to be damaging by SIFT (score = 0.01) and probably damaging by PolyPhen2 (score = 0.997). This variant was in almost complete LD with another missense variant D336N which is not predicted to be damaging. *BTNL2* codes for the butyrophilin like protein 2, which is a member of butyrophilin family that shares sequence homology with the B7 co-stimulatory molecules. The butyrophilins are implicated in T cell inhibition and the modulation of epithelial cell-T cell interactions [22]. *BTNL2* negatively regulates T-cell activation independently of CD28 and CTLA-4, is predominantly expressed in gastrointestinal tissues including human terminal ileum (www.gtexportal.org), and is overexpressed in mouse models of colitis [23]. Recently it has been shown that BTNL2 promotes the expression of Foxp3, which is a transcription factor required for regulatory T cell development and function [24]. In view of its important role in immune modulation and homeostasis and an expression pattern restricted to intestinal epithelial and immune cells, mutations in *BTNL2* may affect its ability to regulate T cell activation in response to mucosal inflammation. Common variants at the *BTNL2* locus, have been previously shown to be associated with ulcerative colitis whilst being independent of the nearby known HLA susceptibility alleles [25]. Additional coding and loss-of-function variants in *BTNL2*, have been associated with susceptibility to other immune related disorders including adult-onset sarcoidosis [26, 27] and rheumatoid arthritis [28].

Although no variants other than the two rare and highly correlated missense mutations in *BTNL2* surpassed the Bonferroni threshold for testing the 79 independent variants for association with IBD, there was significant enrichment for association signals among these 79 variants, and our extension study and combined analysis showed that the direction of the effect for all 5 SNVs tested was consistent with the initial finding. This suggests that there are likely to be additional true positives within phase II and III of our study that have not met the stringent Bonferroni threshold. This emphasises the difficulty in obtaining statistically robust evidence for association of rare variants even with a combined sample of 17,000 tested here and a relatively large effect size such as, for example, *ARIH2* c.338-6C>T, OR = 2.39.

The association of common variants at the *IL12B* locus with both CD and UC is well established [4], although no obvious causal variant has yet been found. The association of the low frequency *IL12B* variant V298F with IBD which was detected in our sequencing experiment was retained in the combined analysis of 10,146 IBD and 7,008 controls, (p = 0.00183, OR = 0.82 [95%CI = 0.72–0.93]). *IL12B* encodes the IL12p40 subunit common to both IL12 and IL23, both of which are produced by activated dendritic cells and macrophages and lead to activation of distinct subsets of T-cells. We found that the minor allele of V298F is associated with a reduced risk of both CD and UC and is independent of the common risk variants at this locus. The variant is predicted to have a damaging or destabilizing effect on protein function or structure, and modeling of the effect of the mutation on the structure of the p40 subunit predicted an altered conformational state which could affect binding to its partner proteins. Thus the rare (Phe) allele may reduce the risk of IBD by attenuating the activation of T cell populations by IL12 and IL23.

We found two additional suggestive associations in *ARIH2* and *NICN1*. Ariadne homolog 2 (*ARIH2*) is a member of an unusual family of E3 ubiquitin-protein ligases. Loss of *ARIH2* has been shown to cause degradation of IκBβ in dendritic cells leading to dysregulated activation of NFκB. The SNP rs200140527 is associated with IBD, and is 6bp upstream of the splice acceptor site for exon 9 of *ARIH2*, although the C>T change is not predicted to affect the strength of the splice site [29]. Nicolin 1 (*NICN*1) is a nuclear protein and part of the neuronal tubulin polyglutamate complex [30] although very little else is known about its function. It is expressed in multiple tissues including the human terminal ileum and transverse colon (www.gtexportal.org). The nonsynonymous SNP p.H191R is associated with a protective effect for CD and UC in this study. *NICN1* is on chromosome 3 at 49.46Mb, i.e. approximately 460kb proximal to *ARIH2* on 3p21 and within a 2Mb locus previously associated with IBD that contains multiple independent genome-wide significant SNPs [4].

Previous sequencing studies have reported that rare coding variants make a limited contribution to the genetics of immune disorders and hypertriglyceridaemia, explaining 1–2% of their genetic variance [10–13, 31, 32]. However, these studies have generally sequenced a limited number of genes located in regions derived from the association of common variants with the disease. Our study highlights the challenges in identifying rare variant association for a polygenic complex trait like IBD. In sequencing more than 500 genes from both GWAS and pathway or network analysis combined with follow up genotyping in over 17,000 individuals we found genome-wide significant association of a rare variant in one gene and suggestive association of 3 SNVs in 3 other genes. However, our follow up studies were powered to detect associations of rare variants with relatively strong effects. For example, our phase II validation panel had 57% power to detect association of a low frequency variant with an allele frequency of 2.5% and OR = 1.3 at alpha level of 0.01 (to flag candidate associations), and 75% power to detect a rare variant with an allele frequency of 1% and OR of 1.6. In the combined analysis of 10,147 cases and 7,008 controls, we had 69% power to confirm association of a variant with a MAF of 0.025 and OR of 1.3 at alpha level of 0.0006 (correction for 79 SNV tests), but 89% power to confirm association for a variant with MAF 0.01 and an OR of 1.6. It is therefore likely that some rare variants with effect sizes of less than 1.6 remain undiscovered in these genes. It is also possible that a proportion of variants that are recognised as being suggestive of association in this study may turn out to be false positives, so further replication and subsequent functional studies will be required to prove causality.

If our 4 newly discovered associations were added to the 26 low frequency SNVs identified in 13 other genes from previously published studies of IBD [7, 10–12, 17, 33] this would total 30 IBD associations with low frequency SNVs in 17 of 548 sequenced genes. However, these screens have predominantly interrogated the coding regions of less than 3% of all known genes. Our study has targeted <25% (198) of all the known genes that map to the 163 IBD associated regions identified by the most recent mapping efforts of the International IBD Consortium [4]. A comprehensive evaluation of the true extent of the contribution of rare coding variants to IBD will have to await whole exome sequencing of very large numbers of case and controls [34], and whole genome sequencing to capture rare regulatory variants in non-coding regions.

The value of studies of rare variants in IBD lies not only in the discovery of additional risk variants which may aid future genetic profiling in at risk populations, but also in their potential to discover further genes and pathways involved in IBD. Our study provides additional evidence of the importance of the regulation of T cell activation and mucosal T cell responses involving BTNL2, and the potential role of proteosomal degradation in the pathogenesis of IBD.

## Materials and Methods

### Selection of candidate genes and design of the capture array

A total of 531 candidate genes were selected based on: (a) Crohn’s disease GWAS hits; (b) GWAS hits from other immune disorders; (c) Pathway analysis based on Gene-set enrichment analysis; (d) IBD related literature; and (e) Network Analysis (Table 1). Details of these selection criteria are provided in S1 Text. Exon coordinates from RefSeq [35] and Ensembl [36] were combined to include all potentially coding regions. Proximal promoters were included by selection of genomic regions from 200 bp upstream to 50 bp downstream of the transcription start site. Putative splice sites were included by addition of five bp each side of coding exons. In total 6,290 genomic intervals were successfully synthesized for the Agilent SureSelect DNA Capture Array. Capture probes (120 bp; 60bp tiling) corresponding to 1,569,003 bp of target sequence.

### Study participants and sample preparation

Crohn’s disease patients for the sequencing experiment (n = 474) were recruited from specialist IBD clinics in London and Newcastle [37] after informed consent and ethical review (REC 05/Q0502/127). Population controls for sequencing (n = 480) were obtained from the 1958 British Birth Cohort [38]. All individuals were of European ancestry. The chances of detecting rare variants with large effects in the sequencing stage was increased by selection of Crohn’s disease (CD) patients with an early age of onset <20 years (n = 204), or with a family history of IBD (n = 174) or both early onset and family history (n = 96). Additionally, 178 (86%) of those individuals with a family history also had at least one affected first degree relative. DNA samples were quantified in triplicate (Qubit, Life technologies) prior to pooling in equimolar amounts to a total of 3 μg of DNA. Pools of 24 CD case DNA samples or 24 control DNA samples were made with a total of 44 pools, 474 cases and 480 controls (including 9 pilot/test pools of 12 and one test pool of 18 CD cases; S1 Text) and libraries were prepared following standard protocols. The validation panel for phase II, consisted of 3,799 unrelated CD and 2,708 unrelated UC, patients recruited by the UK IBD Genetics Consortium [4] and the replication panel consisted of an additional 1644 CD cases and 2018 UC cases recruited from London and Newcastle (as described above). Additional population controls (n[validation] = 3,064; n[replication] = 3622) were from the 1958 British Birth cohort and the National Blood Donor Service [14]. All cases and controls analysed in the replication phase III were independent and unrelated to those sequenced in the phase I and phase II discovery cohort.

### Read alignment and read quality control

Sequencing reads were aligned to the hg18 (NCBI 36) reference genome using Novoalign (version 2.07.09, Novocraft Technologies). We performed quality control using SAM tools [39] and removed PCR duplicates using Picard tools [40]. SNVs and indels were called using SAM-tools and filtered based on the following criteria: i) Phred base quality score ≥ 20, ii) any allele to have at least two base calls on each strand, iii) minimum base call count for any allele to be the equivalent to at least one expected chromosome count (N allele-specific base calls / N total base calls * 2 * N individuals in pool), with at least 0.3 expected chromosome counts attributable to each strand, iiii) criteria to be met in at least three different pools from at least two different batches. These parameters were optimized to reduce biases across all 44 pools (S2 Fig.). After filtering, base call counts were normalised to allele frequencies for each pool based on the total number of base calls that passed the filtering criteria. Variants were annotated using ANNOVAR [41]. Further details of read alignment, quality control and variant calling are provided in S1 Text.

### Validation genotyping and extension study

After excluding variants previously implicated with IBD and variants analysed in the IBD Immunochip project [4], we selected 96 SNVs for follow up in phase II using the Sequenom iplex genotyping platform. We chose variants that a) surpassed multiple testing in the pooled sequencing based case-control comparison (p < 10^−5^), b) were modestly significant in the pooled sequencing based case-control comparison (p < 0.05) and had a low allele frequency (MAF < 5%), c) had functional consequence (within 20bp of a splice acceptor or donor site or non-synonymous variant), and were novel or low frequency (< 1%), d) were absent from one group (either controls or cases) and had a functional consequence (within 20bp of a splice acceptor or donor site or non-synonymous variant). In total 84 SNVs passed design and were genotyped via Sequenom iplex in 2,974 controls, 3,715 Crohn’s disease and 2,620 ulcerative colitis cases. Individuals for which more than 20% of SNVs could not be called were excluded from further analysis. One additional SNV (rs138274580/ATG4B) and 3 indels (rs58682836/COBL frameshift delTTC, rs71297581/TYK2 upstream insC, rs3833864/PIK3C upstream insC), that failed iplex design, were genotyped using the TaqMan chemistry (Life Technologies); SNP since they were ranked as high priority in all categories of our variant selection criteria (S1 Text). Finally we selected 5 SNVs with p<0.01 in any one phenotype (CD, UC or IBD) and, in the case of multiple SNVs in *BTNL2*, were indicated by LD and conditional regression analysis to be independent of each other and the known common risk variants, for replication genotyping via KASP^TM^ chemistry at LGC Genomics (Hoddesdon, Herts, UK) in 3666 additional IBD cases and 3622 additional controls. In order to validate previous phases we also included a further 858 individuals who had been sequenced and/or undergone sequenom iplex genotyping. To investigate LD and independence of the *BTNL2* variants from the known IBD GWAS hits within the MHC we used Immunochip data supplied by the UKIBD Genetics Consortium that was available for 1,638 of our genotyped IBD cases and 1,243 of genotyped controls.

### Statistical analyses

Allele frequencies for each SNV in each pool were standardized and subjected to principal components analysis (PCA) to identify outlier pools and investigate systematic bias between cases and controls. PCA revealed considerable bias, such that cases and control pools could be largely separated by PC axis 1 alone. Various statistical methods for dealing with pooled SNV data have been proposed [16]. In light of the PCA results, we adopted a genomic control approach because it can correct for both overdispersion (additional variance that is distributed equally among pools) and bias (a consistent tendency for allele frequencies in case pools to be different from controls pools). For each SNV, a logistic regression across pools was performed using expected chromosome counts for the two most common alleles to form the dependent variable, and case-control status as the independent variable. The reversal of the conventional functional from allows for different pool sizes to be readily accounted for, and also appropriately reflects the study design (pool status is fixed by the experimenter, not pool allele frequencies). Genomic control was performed by dividing the chi-square statistic for association by the median chi-square statistic across all SNVs. We used evidence for especially strong SNV-specific overdispersion among pools (via a test of residual deviance from the logistic regression for association, p < 1.5x10^−5^) as an additional QC measure for removal of suspect SNVs.

Burden tests for significant association of a group of SNVs (e.g. all SNVs in a gene) were also performed taking in account both the pooled design and the presence of case-control bias. For a given set of n SNVs, genomic-control-corrected z^2^ values were summed and tested against the chi-squared distribution with n degrees of freedom. Significant sum-statistics were further tested via permutation of case-control status among pools, to correct for false positives that could be caused from linkage disequilibrium distributing the same signal among multiple SNVs. Note that our burden test allows SNV groups containing a mixture of both risk and protective variants to be tested appropriately.

Statistical analyses of pooled sequencing data was performed using R project for statistical computing (http://www.r-project.org/). Cases-control analysis of validation and replication genotyping data was performed with PLINK version 1.07 [42] using Armitage Trend Test. Additional conditional regression, linkage disequilibrium and haplotype analysis at known common IBD loci was performed using UNPHASED v3.0.12 [43].

### Structural analysis of IL12B

The effect of the mutation Val298Phe on IL12B (p40) protein stability was examined using the tool mCSM, which predicts the effect of mutations in proteins using graph-based signatures [20]. The structure of the complex of human IL12B (p40) and IL23A (p19) is available in the RCSB Protein Data Bank [44] (PDB entry 4GRW), and was used as the template to model the structure of mutant IL12B^V298F^. The modelling procedure first generated the sequence alignment between the target (IL12B^V298F^) and the template structure (4GRW chain B) by running the tool T-Coffee [45]. The aligned sequences were then used as an input to the structure modelling package Modeller 9v8 [46]to generate 200 structures of IL12B^V298F^. Among these, only the one with the best Discrete Optimized Protein Energy score was selected for inspection of the mutation Val298Phe. The structure representation tool PyMol (Version 1.5.0.4, Schrödinger, LLC)was used for visual inspection and structural analysis. The interaction between IL12B^V298F^ and IL23A was modelled by superimposing the IL12B^V298F^ structure onto the human wild-type IL12B.

## Supporting Information

S1 Text Supplementary Materials and Methods and additional supplementary references. (DOCX)

S1 Fig Total number of variants and coverage across all 44 pools. The total number of variants identified is dependent on the coverage and the number of individuals per pool.
(TIF)

S2 Fig Types of SNVs per pool after filtering. The number of different types of SNV are compared across 44 pools. SNVs are only considered if they pass filtering criteria as described in Materials and Methods. Numbers are approximately constant across pools 11–44 whilst lower numbers are observed for earlier pools.
(TIF)

S3 Fig A plot of the first two principal component (PC) axes derived from allele frequencies estimated by the sequencing data after removing pool numbers 7 and 8. (TIF)

S4 Fig Structural analysis of the IL12B mutation V298F. Comparison between IL12B^wt^ (blue) and IL12B^V298F^ (green) and their interaction with IL23A (grey). The variant Val298Phe is located in the Fibronectin III (FN3) domain of IL12B, and is shown with a stick representation, coloured in magenta for IL12B^V298F^ and red for IL12B^wt^. A close up of the FN3 domain is given on the right of the figure. The neighbouring β-sheets (coloured in teal) were shortened in the modelled structure of IL12B ^V298F^ compared to IL12B^wt^. The loops 5 and 6 (coloured in magenta and orange) are important for the binding of p40 to partner proteins IL23A (p19) and IL12A (p35), thus the altered conformational state of this region of the molecule could affect optimal binding to these partners.
(TIF)

S1 Table A table showing all SNVs and insertion deletions that were identified in the discovery sequencing (phase I) and passed filtering (Materials and Methods). (XLSX)

S2 Table Known rare and common CD risk variants identified by our pooled sequencing strategy. (DOCX)

S3 Table Top 20 genes from the gene burden analysis of predicted functional variants showing the number of functional variant per gene, the sum of the z2 statistic and the p-value for association with CD. (DOCX)

S4 Table All 80 SNPs that were selected for validation in phase II. The adjusted p-values and residual deviance obtained in the pooled sequencing analysis is shown, along with location with respect to the nearby gene and the effect on the primary protein structure for coding SNPs.
(DOCX)

S5 Table Sequence variants associated (p<0.05) with either Crohn’s disease, ulcerative colitis or inflammatory bowel disease in the Phase II validation study (XLSX)

S6 Table Single variant and conditional analysis of rare BTNL2 variants and common UC risk and CD GWAS variants in 1638 IBD cases and 1243 controls. (DOCX)

S7 Table Haplotype analysis in 1638 IBD cases and 1243 controls for rare *BTNL2* variant p.G454C (rs28362675), and common UC GWAS variant (rs477515) (DOCX)
