# Correction: Pooled Sequencing of 531 Genes in Inflammatory Bowel Disease Identifies an Associated Rare Variant in *BTNL2* and Implicates Other Immune Related Genes

**DOI:** 10.1371/journal.pgen.1005172

**Published:** 2015-04-17

**Authors:** The PLOS Genetics Staff

The UK IBD Genetics Consortium is listed out of order in the author list. The correct order is:

Natalie J. Prescott^1‡^ *, Benjamin Lehne^1,2‡^, Kristina Stone^1^, James C. Lee^3^, Kirstin Taylor^1^, Jo Knight^1,4,5^, Efterpi Papouli^6^, Muddassar M. Mirza^6^, Michael A. Simpson^1^, Sarah L. Spain^1^, Grace Lu^7^, Franca Fraternali^7^, Suzannah J. Bumpstead^8^, Emma Gray^8^, Ariella Amar^1^, Hannah Bye^1^, Peter Green^1^, UK IBD Genetics Consortium^¶^, Guy Chung-Faye^9^, Bu’Hussain Hayee^9^, Richard Pollok^10^, Jack Satsangi^11^, Miles Parkes^3^, Jeffrey C. Barrett^8^, John C. Mansfield^12^, Jeremy Sanderson^13^, Cathryn M. Lewis^1^, Michael E. Weale^1‡^, Thomas Schlitt^1‡^, Christopher G. Mathew^1,14‡^

**1** Department of Medical and Molecular Genetics, Kings College London, Guy’s Hospital, London, United Kingdom, **2** Department of Epidemiology and Biostatistics, Imperial College London, London, United Kingdom, **3** Department of Medicine, University of Cambridge School of Clinical Medicine, Cambridge Biomedical Campus, Cambridge, United Kingdom, **4** Campbell Family Mental Health Research Institute, Centre for Addiction and Mental Health, Toronto, Ontario, Canada, **5** Department of Psychiatry, University of Toronto, Toronto, Ontario, Canada, **6** NIHR GSTFT/KCL Comprehensive Biomedical Research Centre Genomics Core Facility, King’s College London School of Medicine, Guy’s Hospital, London, United Kingdom, **7** Randall Division of Cell and Molecular Biophysics, King’s College London, Guy’s Campus, London, United Kingdom, **8** Wellcome Trust Sanger Institute, Wellcome Trust Genome Campus, Hinxton, Cambridge, United Kingdom, **9** Department of Gastroenterology, King’s College Hospital NHS Foundation Trust, Denmark Hill, London, United Kingdom, **10** Gastroenterology and Hepatology, St George’s Healthcare NHS Trust, Tooting, London, United Kingdom, **11** Gastrointestinal Unit, Molecular Medicine Centre, University of Edinburgh, Western General Hospital, Edinburgh, United Kingdom, **12** Institute of Genetic Medicine, Newcastle University, Newcastle upon Tyne, United Kingdom, **13** Guy’s & St Thomas’ NHS Foundation Trust, St Thomas’ Hospital, Department of Gastroenterology, London, United Kingdom, **14** Division of Medical Biochemistry, University of Cape Town, Cape Town, South Africa

‡ NJP and BL contributed equally to this work. MEW, TS, and CGM also contributed equally to this work.

¶ Membership of the UK IBD Genetics Consortium is listed in the Acknowledgments.

* E-mail: natalie.prescott@kcl.ac.uk
