# Supplementary material for: Pooled Sequencing of 531 Genes in Inflammatory Bowel Disease Identifies an Associated Rare Variant in BTNL2 and Implicates Other Immune Related Genes
Source: PLoS Genet. 2015 Feb 11;11(2):e1004955. doi: 10.1371/journal.pgen.1004955 (PMC4335459; doi:10.1371/journal.pgen.1004955)
Supplement: S1 Text — (DOCX) [file pgen.1004955.s001.docx]

# Text S1: Supplementary Methods

##### Selecting Candidate Genes

In total 531 candidate genes were selected using five different strategies. The genes selected for each strategy are non-exclusive as many genes were selected by more than one strategy.

##### Genes Based on Crohn’s Disease Related Literature

We selected genes based on the literature of Crohn’s disease. This includes all genes in loci that showed genome-wide association with CD as of January 2010 [[51](#_ENREF_1), [52](#_ENREF_2)]. We also included genes reviewed by Weersma *et al.* [[52](#_ENREF_2)] whose association had been reported elsewhere and genes implicated in the IL12/IL23 pathway [[53](#_ENREF_3)]. In addition several genes were selected manually via literature searches based on their involvement in the autophagy pathway. This resulted in a total of 214 genes.

##### Genes Found by Genome-wide Association Studies on Crohn’s Disease

Based on an early meta-analysis of Crohn’s disease [[51](#_ENREF_1)] we selected all genes where at least one of the 500 most associated SNPs is located less than 40 kb from the transcribed region, which resulted in 75 genes.

##### Genes Found by Genome-wide Association Studies on Auto-immune Diseases

Genes associated with autoimmune disorders were selected using the NHGRI GWAS Catalogue [[54](#_ENREF_4)] in August 2009. We selected genes that showed association (p<10^-11^) with Ulcerative Colitis, Rheumatoid Arthritis, Multiple Sclerosis, Systemic Lupus Erythematosus, Psoriasis, Type 1 Diabetes, Celiac Disease, Crohn’s disease or Inflammatory Bowel Disease. In total 50 genes were selected based on GWAS of autoimmune disorders.

##### Genes Identified Using Gene Set Enrichment Analysis

We performed Gene Set Enrichment Analysis [[55](#_ENREF_5)] based on gene-sets that represent molecular pathways. Gene Sets were obtained from the Molecular Signatures Database [[56](#_ENREF_6)] in August 2009. Using a hyper-geometric test we looked for overrepresentation of the Crohn’s disease Literature genes (see above). In total 65 pathways containing 74 genes showed statistically significant enrichment.

##### Genes Identified Using Network Analyses

Candidate genes were selected based on the analysis of three Protein Interaction Networks (PINs). As described previously, we integrated experimentally measured protein-protein interactions from six public databases [[57](#_ENREF_7)] and derived PINs based on interactions that were reported by at least one (HuPPI) and at least two (HuPPI2) independent publications. A third network, termed HuSTR700, is based on the STRING database which combines protein-protein interaction with additional data sources such as co-citation in Pubmed abstracts and co-expression across different tissues [[58](#_ENREF_8)]. HuSTR700 was derived by retaining all interactions in STRING that had a confidence score above 0.7 which results in a network with 11,262 nodes and 301,175 edges. We performed Markov Clustering [[59](#_ENREF_9)] on all three PINs which resulted in 38 clusters for HuPPI, 29 clusters for HuPPI2 and 40 clusters for the HuSTR700. All clusters were tested for enrichment of the literature genes using a hyper-geometric test resulting in 8 clusters that show statistically significant enrichment (2 HuPPI, 2 HuPPI2, 4 HuSTR700). In total the 8 clusters include 215 genes, all of which were selected.

In addition all clusters were analysed using an approach termed Region Growing Analysis (RGA) which has been described previously [[60](#_ENREF_10)]. In brief, gene-wide p-values were derived from GWAS data for Crohn’s Disease provided by the Wellcome Trust Case Control Consortium [[61](#_ENREF_11)] as described previously [[62](#_ENREF_12)]. Based on these gene-wide p-values, RGA searches for connected subnetworks that contain genes with nominally significant p-values (p<0.05) and allows node “jumps” of maximally one additional gene previously [[60](#_ENREF_10)]. Using this approach we selected 134 genes. In total we selected 300 genes by network analyses. Combining the five strategies results in a total of 531 genes (multiple genes were selected by more than one strategy (**Table 1**)

##### Read Alignment and Read Quality Control

Sequencing reads were aligned to the hg18 (NCBI 36) reference genome using Novoalign (version 2.07.09, Novocraft Technologies). Prior to alignment Illumina adapter sequences, reads consisting of homo-polymers and reads with low overall base-qualities are removed based on novoalign default settings (-a flag; homopolymer score > 20; minimum number of good quality bases per read > log4(length reference genome)+5). Base qualities are recalibrated based on the proportion of nucleotide mismatches with the reference sequence as a function of the corresponding position in the sequencing read, the reported base quality score and other properties of the mismatched base. In the case of nucleotide mismatches at the 3’ end of a sequencing read, the read is clipped to the optimal alignment (soft-clip). Insertions and deletions (indels) in coding regions have the propensity to be multiples of 3 nucleotides. To take this into account and accurately align indels in coding sequences, values to penalize gap opening and gap extension are changed to 65 and 7, respectively. Only the best alignment for reads that map to multiple locations is retained. Using SAM (Sequence Alignment/Map) tools version 0.1.13 [[63](#_ENREF_13)] we performed a local realignment of all reads and discarded reads with a mapping quality below 1. PCR duplicates of unpaired reads and read pairs were removed using Picard tools version 1.40 [[64](#_ENREF_14)].

We defined the capture efficiency as the proportion of post quality control reads that overlap by at least one base pair with the genomic intervals corresponding to the Agilent SureSelect capture probe sequences. The coverage is defined as the number of post quality control reads that cover each base pair of the genomic intervals of the Agilent SureSelect capture probe sequences. The capture efficiency and the coverage was calculated using BED (Browser Extensible Data) tools [[65](#_ENREF_15)].

##### Variant Calling and Variant Filtering

We considered both single nucleotide variants (SNVs) and insertions and deletions (indels) in this analysis. Existing variant callers for NGS data assume a single diploid genome and are not suitable for pooled sequencing data. We therefore developed a new protocol to analyse NGS data generated from DNA pools. We used mpileup (samtools version 0.1.13) [[63](#_ENREF_13)] to generate a mpileup file which contains base calls for each pool and position of the capture array. Using a custom-made python script the mpileup file is parsed and base calls are counted for each pool and genomic position. When considering all variant calls, we observed an overrepresentation of C>A and G>T transversions compared to all other types of variants. Although at low frequencies (<2.5%) in our data, this imbalance has been reported previously [10]. Momozawa *et al.* traced their origin to a clonal PCR step, suggesting that they arise as the consequence of a base mis-incorporation during the library preparation. In order to correct for this bias and to reduce false positive calls due to sequencing errors or contamination, base calls were filtered based on the following criteria:

1. Phred base quality score ≥ 20 [[66](#_ENREF_16), [67](#_ENREF_17)].
2. Any allele must have at least two base calls on each strand
3. Mminimal base call count for any allele has to be the equivalent of at least one allele:

$$minimal base call count \geq1.0\times\frac{total base calls}{alleles in pool}$$

4. Minimal base call count of any allele per strand has to be the equivalent of at least 0.3 allele:

$$minimal base call count per strand \geq0.3\times\frac{total base calls}{alleles in pool}$$

5. Any allele must pass the first four criteria in at least three different pools from at least two different runs

When these criteria were met the corresponding base calls were considered to be true. Base calls that did not meet these criteria were dropped. A variant is called when a filtered base call differs from the reference sequence. Base calls counts are normalised to allele frequencies for each pool based on the total number of base calls that pass the filtering criteria. After filtering, the normalised number of of C>A and G>T transversions was approximately constant across all pools as were all other types of variants (**Figure S2**).

For a small number of variants only a single allele was observed in all pools. These variants were still called because the respective allele differs from the reference sequence. These “mono-allelic” variant-calls likely to represent putative errors in the reference sequence and were excluded from further analyses.

# Supporting references

51. Barrett JC, Hansoul S, Nicolae DL, Cho JH, Duerr RH, et al, (2008) Genome-wide association defines more than 30 distinct susceptibility loci for Crohn's disease. Nat. Genet. 40: 955-62.

52. Weersma RK, Stokkers PC, Cleynen I, Wolfkamp SC, Henckaerts L, et al, (2009) Confirmation of multiple Crohn's disease susceptibility loci in a large Dutch-Belgian cohort. Am J Gastroenterol 104: 630-8.

53. Wang K, Zhang H, Kugathasan S, Annese V, Bradfield JP, et al, (2009) Diverse genome-wide association studies associate the IL12/IL23 pathway with Crohn Disease. Am J Hum Genet 84: 399-405.

54. Hindorff LA, Junkins HA, Hall PN, Mehta JP, and Manolio TA. *A Catalog of Published Genome-Wide Association Studies*. August 2009 [cited 2009 26.08.2009]; Available from: [www.genome.gov/gwastudies](http://www.genome.gov/gwastudies).

55. Mootha VK, Lindgren CM, Eriksson KF, Subramanian A, Sihag S, et al, (2003) PGC-1alpha-responsive genes involved in oxidative phosphorylation are coordinately downregulated in human diabetes. Nat Genet 34: 267-73.

56. Subramanian A, Tamayo P, Mootha VK, Mukherjee S, Ebert BL, et al, (2005) Gene set enrichment analysis: a knowledge-based approach for interpreting genome-wide expression profiles. Proc Natl Acad Sci U S A 102: 15545-50.

57. Lehne B and Schlitt T, (2009) Protein-protein interaction databases: keeping up with growing interactomes. Hum Genomics 3: 291-7.

58. Jensen LJ, Kuhn M, Stark M, Chaffron S, Creevey C, et al, (2009) STRING 8--a global view on proteins and their functional interactions in 630 organisms. Nucleic Acids Res 37: D412-6.

59. Dongen Sv, Graph Clustering by Flow Simulation, 2000, Bioinformatics, University of Utrecht.

60. Lehne B, Computational Analyses of Complex Diseases at the Gene and Network Levels, 2012, Doctoral Thesis, Department of Medical and Molecular Genetics King's College London.

61. Wellcome Trust Case Control Consortium, (2007) Genome-wide association study of 14,000 cases of seven common diseases and 3,000 shared controls. Nature 447: 661-678.

62. Lehne B, Lewis CM, and Schlitt T, (2011) From SNPs to Genes: Disease Association at the Gene Level. PLoS One 6: e20133.

63. Li H, Handsaker B, Wysoker A, Fennell T, Ruan J, et al, (2009) The Sequence Alignment/Map format and SAMtools. Bioinformatics 25: 2078-9.

64. Wysoker A, Tibbetts K, Fennell T, and Weisburd B, Picard Tools, 2009.

65. Quinlan AR and Hall IM, (2010) BEDTools: a flexible suite of utilities for comparing genomic features. Bioinformatics 26: 841-2.

66. Ewing B, Hillier L, Wendl MC, and Green P, (1998) Base-calling of automated sequencer traces using phred. I. Accuracy assessment. Genome Res 8: 175-85.

67. Ewing B and Green P, (1998) Base-calling of automated sequencer traces using phred. II. Error probabilities. Genome Res 8: 186-94.
