# Supplementary figures and images for: Pooled Sequencing of 531 Genes in Inflammatory Bowel Disease Identifies an Associated Rare Variant in BTNL2 and Implicates Other Immune Related Genes
Source: PLoS Genet. 2015 Feb 11;11(2):e1004955. doi: 10.1371/journal.pgen.1004955 (PMC4335459; doi:10.1371/journal.pgen.1004955)

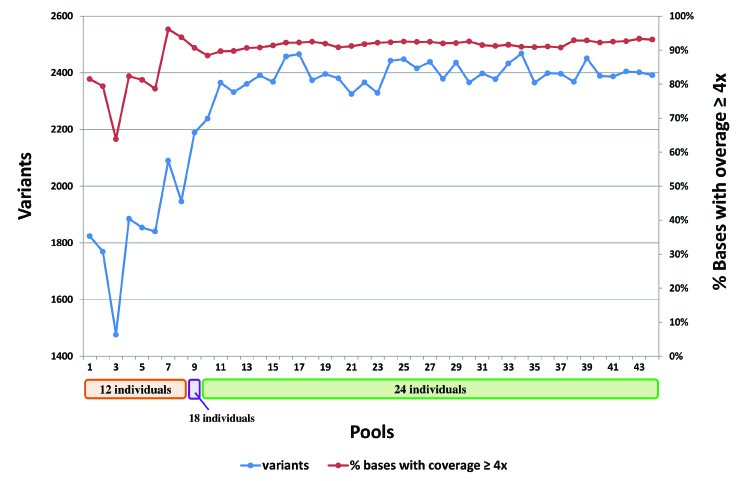

Supplement: S1 Fig — The total number of variants identified is dependent on the coverage and the number of individuals per pool. (TIF) [file pgen.1004955.s002.tif]

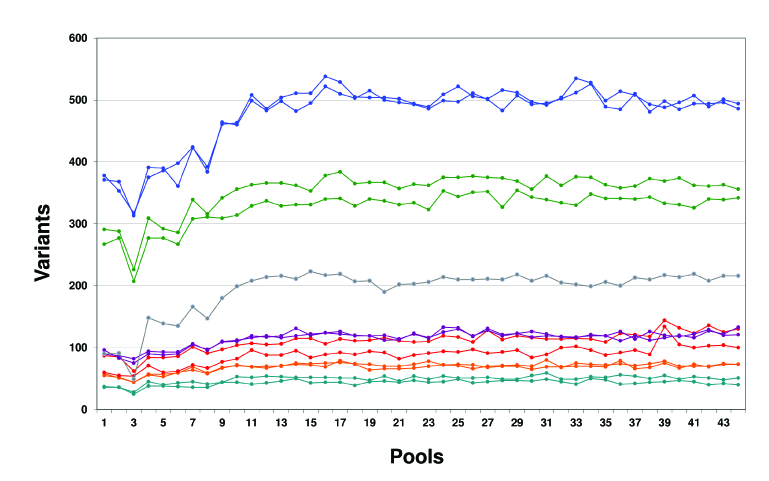

Supplement: S2 Fig — The number of different types of SNV are compared across 44 pools. SNVs are only considered if they pass filtering criteria as described in Materials and Methods. Numbers are approximately constant across pools 11–44 whilst lower numbers are observed for earlier pools. (TIF) [file pgen.1004955.s003.tif]

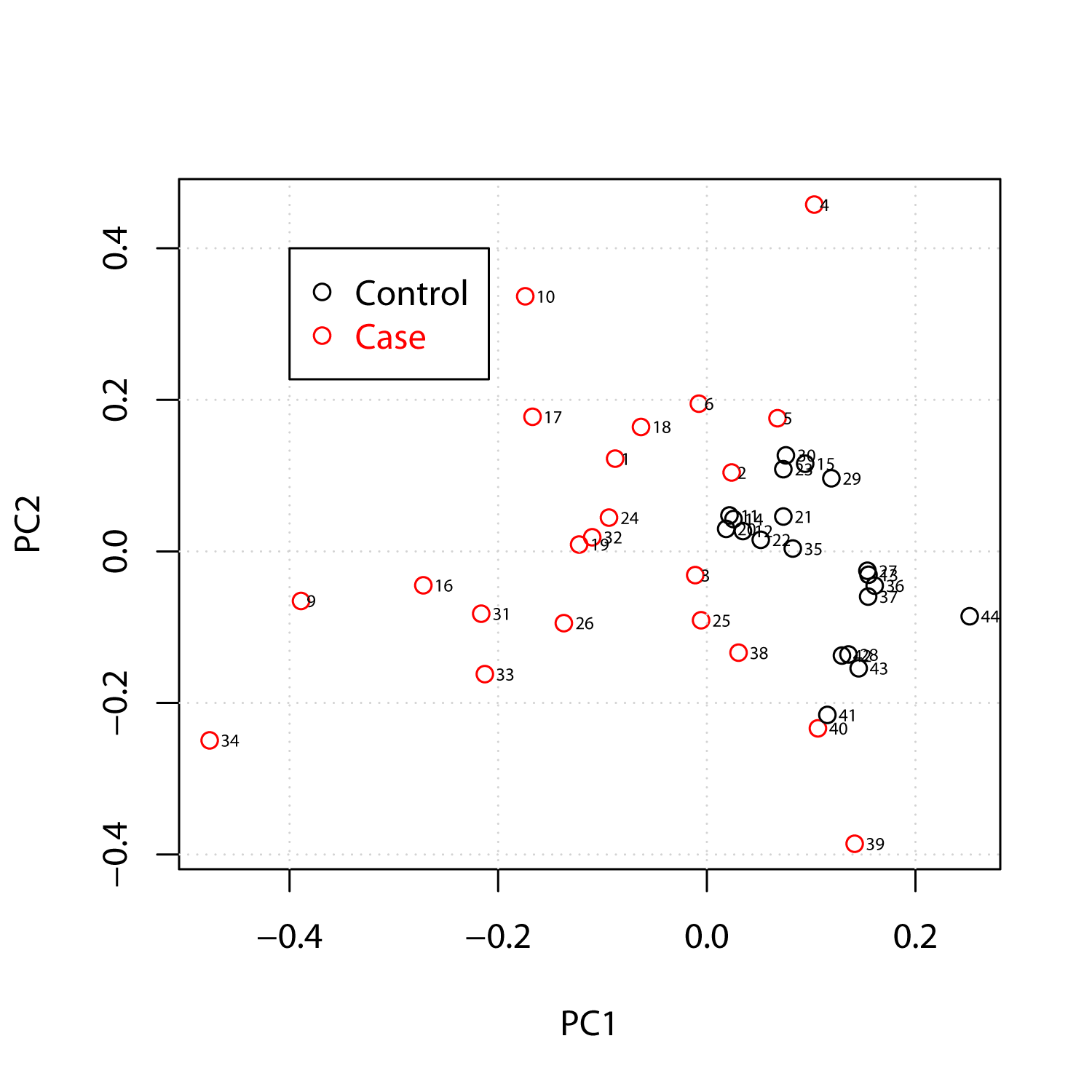

Supplement: S3 Fig — (TIF) [file pgen.1004955.s004.tif]

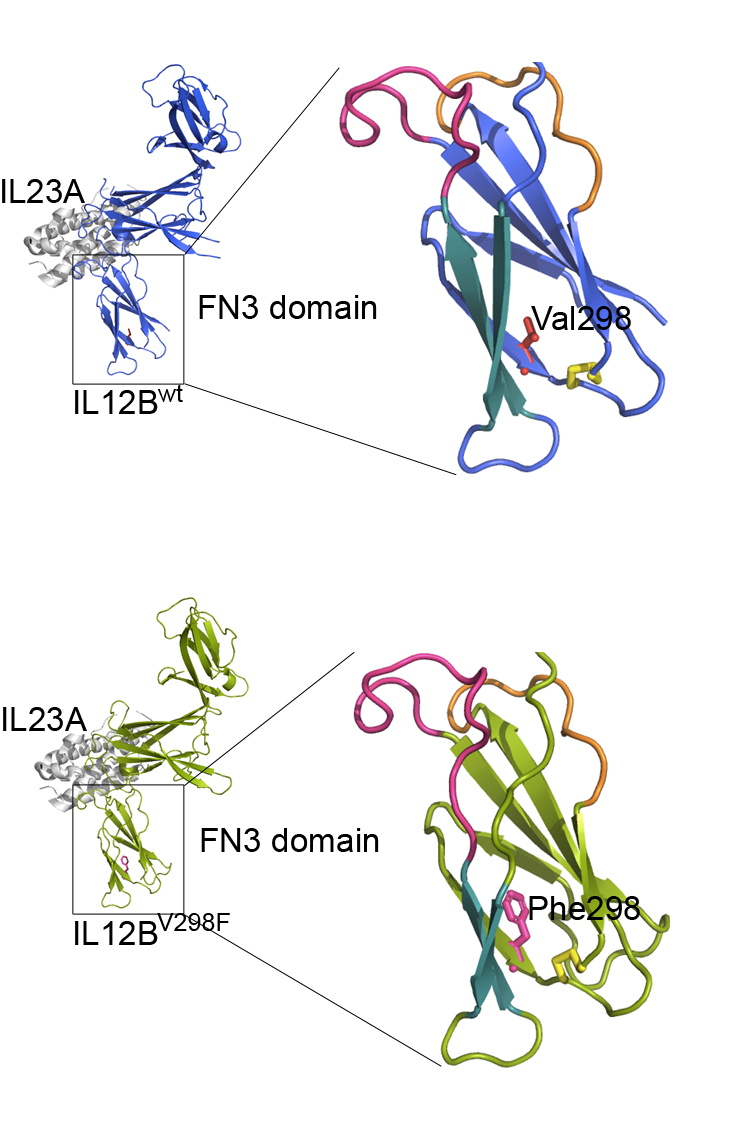

Supplement: S4 Fig — Comparison between IL12Bwt (blue) and IL12BV298F (green) and their interaction with IL23A (grey). The variant Val298Phe is located in the Fibronectin III (FN3) domain of IL12B, and is shown with a stick representation, coloured in magenta for IL12BV298F and red for IL12Bwt. A close up of the FN3 domain is given on the right of the figure. The neighbouring β-sheets (coloured in teal) were shortened in the modelled structure of IL12B V298F compared to IL12Bwt. The loops 5 and 6 (coloured in magenta and orange) are important for the binding of p40 to partner proteins IL23A (p19) and IL12A (p35), thus the altered conformational state of this region of the molecule could affect optimal binding to these partners. (TIF) [file pgen.1004955.s005.tif]
