# Supplementary material for: Pooled Sequencing of 531 Genes in Inflammatory Bowel Disease Identifies an Associated Rare Variant in BTNL2 and Implicates Other Immune Related Genes
Source: PLoS Genet. 2015 Feb 11;11(2):e1004955. doi: 10.1371/journal.pgen.1004955 (PMC4335459; doi:10.1371/journal.pgen.1004955)
Supplement: S2 Table — (DOCX) [file pgen.1004955.s007.docx]

**Table S2**

| **SNP** | **gene** | **Disease variant** | **PublishedMAF CD** | **PublishedOR** | **Seq MAF CD** | **Seq MAF Cntrl** | **Seq P val** | **Seq OR** |
| --- | --- | --- | --- | --- | --- | --- | --- | --- |
| rs2241880 | *ATG16L1* | p.T300A | 58.10% | 1.31^a^ | 53.80% | 47.20% | 7.71E-05 | 1.45 |
| rs76418789 | *IL23R* | p.G149R | 0.25% | 0.6^b^ | 0.16% | 0.39% | 0.884294 | 0.41 |
| rs41313262 | *IL23R* | p.V362I | 1.10% | 0.72^b^ | 0.62% | 1.39% | 0.139816 | 0.44 |
| rs11209026 | *IL23R* | p.R381Q | 3.00% | 0.36^c^ | 1.90% | 5.70% | 0.000567 | 0.31 |
| rs10065172 | *IRGM* | p.L105L | 10.70% | 1.56^d^ | 10.50% | 5.70% | 0.0004 | 1.92 |
| rs2066844 | *NOD2* | p.R702W | 4.10% | 2.2^e^ | 9.40% | 3.10% | 6.31x10^-8^ | 3.25 |
| rs5743277 | *NOD2* | p.R703C | 0.79% | 1.51^b^ | 0.70% | 0.31% | 0.335577 | 2.31 |
| rs2066845 | *NOD2* | p.G908R | 1.50% | 2.99^e^ | 2.30% | 0.70% | 0.0078 | 3.53 |
| s41450053 | *NOD2* | p.L1007fs | 1.90% | 4.09^e^ | 4.70% | 0.40% | 2.74E-06 | 2.51 |

The observed minor allele frequencies (MAF) of 9 known CD susceptibility variants, their previously described MAF and odds ratios.

a [[1](#_ENREF_1)]; b [[2](#_ENREF_2)]; c [[3](#_ENREF_3)]; d [[4](#_ENREF_4)]; e [[5](#_ENREF_5)]

1. Prescott NJ, Fisher SA, Franke A, Hampe J, Onnie CM, et al, (2007) A Nonsynonymous SNP in ATG16L1 Predisposes to Ileal Crohn's Disease and Is Independent of CARD15 and IBD5. Gastroenterology 132: 1665-1671.

2. Rivas MA, Beaudoin M, Gardet A, Stevens C, Sharma Y, et al, (2011) Deep resequencing of GWAS loci identifies independent rare variants associated with inflammatory bowel disease. Nat Genet 43: 1066-73.

3. Momozawa Y, Mni M, Nakamura K, Coppieters W, Almer S, et al, (2011) Resequencing of positional candidates identifies low frequency IL23R coding variants protecting against inflammatory bowel disease. Nat Genet 43: 43-7.

4. Prescott NJ, Dominy KM, Kubo M, Lewis CM, Fisher SA, et al, (2010) Independent and population-specific association of risk variants at the IRGM locus with Crohn's disease. Hum Mol Genet 19: 1828-39.

5. Economou M, Trikalinos TA, Loizou KT, Tsianos EV, and Ioannidis JP, (2004) Differential effects of NOD2 variants on Crohn's disease risk and phenotype in diverse populations: a metaanalysis. Am J Gastroenterol 99: 2393-404.
