# Supplementary material for: Pooled Sequencing of 531 Genes in Inflammatory Bowel Disease Identifies an Associated Rare Variant in BTNL2 and Implicates Other Immune Related Genes
Source: PLoS Genet. 2015 Feb 11;11(2):e1004955. doi: 10.1371/journal.pgen.1004955 (PMC4335459; doi:10.1371/journal.pgen.1004955)
Supplement: S3 Table — (DOCX) [file pgen.1004955.s008.docx]

**Table S3**

| **gene** | **n(variants)** | **sum z2** | **p-value** |
| --- | --- | --- | --- |
| NOD2 | 10 | 41.54564 | 9.03E-06 |
| BTNL2 | 18 | 49.78091 | 8.15E-05 |
| MIER1 | 3 | 19.69008 | 1.97E-04 |
| ATG16L1 | 1 | 12.59071 | 3.88E-04 |
| SAA2 | 5 | 18.66794 | 2.22E-03 |
| EBI3 | 1 | 7.970831 | 4.75E-03 |
| PTPRC | 4 | 13.16113 | 1.05E-02 |
| PDE4A | 1 | 6.461718 | 1.10E-02 |
| POPDC3 | 1 | 6.098482 | 1.35E-02 |
| IL1RL1 | 9 | 20.70073 | 1.40E-02 |
| CARD9 | 3 | 10.59343 | 1.41E-02 |
| CCL4 | 1 | 5.87693 | 1.53E-02 |
| CCL25 | 4 | 11.58029 | 2.08E-02 |
| IL12B | 1 | 5.226864 | 2.22E-02 |
| C1orf141 | 4 | 10.96806 | 2.69E-02 |
| LCE3D | 1 | 4.895134 | 2.69E-02 |
| PREP | 2 | 6.809441 | 3.32E-02 |
| ZNF572 | 4 | 10.38018 | 3.45E-02 |
| DALRD3 | 1 | 4.322563 | 3.76E-02 |
| ATG7 | 1 | 4.319236 | 3.77E-02 |
