# Supplementary material for: Pooled Sequencing of 531 Genes in Inflammatory Bowel Disease Identifies an Associated Rare Variant in BTNL2 and Implicates Other Immune Related Genes
Source: PLoS Genet. 2015 Feb 11;11(2):e1004955. doi: 10.1371/journal.pgen.1004955 (PMC4335459; doi:10.1371/journal.pgen.1004955)
Supplement: S4 Table — The adjusted p-values and residual deviance obtained in the pooled sequencing analysis is shown, along with location with respect to the nearby gene and the effect on the primary protein structure for coding SNPs. (DOCX) [file pgen.1004955.s009.docx]

**Table S4**

| **chrom** | **Pos (hg19)** | **Maj allele** | **Min allele** | **hgnc symbol** | **SNP ID** | **location** | **coding SNP?** | **consequence per transcript** | **p value (adj)** | **Freq CD** | **Freq Cntrl** | **Rdev** |
| --- | --- | --- | --- | --- | --- | --- | --- | --- | --- | --- | --- | --- |
| 1 | 20208706 | T | C | OTUD3 | TC_1_20081293 | upstream | . |  | 0.093409262 | 0.0086 | 0.0154 | 22.38 |
| 1 | 57158051 | G | A | PRKAA2 | rs17848595 | exonic | sSNP | p.R117R | 0.008351849 | 0.0557 | 0.0341 | 28.97 |
| 1 | 63284830 | G | A | ATG4C | rs17123872 | exonic | sSNP | p.G183G | 0.011405661 | 0.0231 | 0.0496 | 26.06 |
| 1 | 117113859 | C | T | CD58 | CT_1_116915382 | upstream | . |  | 0.220877858 | 0.0032 | 0.0074 | 24.19 |
| 1 | 153606479 | G | A | CHTOP | GA_1_151873103 | UTR5 | . |  | 0.045280611 | 0.0116 | 0.0249 | 40.85 |
| 1 | 153614712 | C | T | CHTOP | rs7549308 | splicing | . |  | 0.001052027 | 0.0349 | 0.0140 | 48.32 |
| 1 | 198510045 | G | A | ATP6V1G3 | rs943132 | UTR5 | . |  | 0.10270981 | 0.0070 | 0.0129 | 24.65 |
| 1 | 198691595 | T | A | PTPRC | rs12136658 | exonic | nsSNP | p.H407Q (NM_080921); p.H568Q (NM_002838) | 0.026577626 | 0.0263 | 0.0137 | 36.31 |
| 2 | 24920663 | A | G | NCOA1 | rs41281513 | exonic | sSNP | p.Q315Q | 0.046320779 | 0.0223 | 0.0098 | 32.42 |
| 2 | 24930262 | C | T | NCOA1 | rs41281515 | exonic | sSNP | p.A641A | 0.049755511 | 0.0166 | 0.0060 | 36.81 |
| 2 | 32449832 | C | A | NLRC4 | rs61754192 | exonic | nsSNP | p.A929S | 0.063546669 | 0.0076 | 0.0182 | 30.75 |
| 2 | 70475680 | C | T | TIA1 | CT_2_70329184 | UTR5 | . |  | 0.212019735 | 0.0000 | 0.0045 | 14.20 |
| 2 | 71063111 | C | T | CD207 | rs72836224 | upstream | . |  | 0.036597959 | 0.0100 | 0.0220 | 33.22 |
| 2 | 102851470 | G | A | IL1RL2 | rs75091099 | exonic | nsSNP | p.A471T | 0.348241366 | 0.0015 | 0.0061 | 24.97 |
| 2 | 102956715 | C | T | IL1RL1 | CT_2_102323147 | exonic | nsSNP | p.P144S | 0.996324922 | 0.0074 | 0.0000 | 12.75 |
| 2 | 102958718 | G | A | IL1RL1 | rs111970215 | exonic | nsSNP | p.A216T | 0.129538344 | 0.0014 | 0.0061 | 20.74 |
| 2 | 113820124 | C | T | IL1F5 | CT_2_113536595 | exonic | nsSNP | p.S113L | 0.705583983 | 0.0058 | 0.0065 | 25.58 |
| 2 | 153519524 | G | A | PRPF40A | rs1036526 | intronic | . |  | 0.020579538 | 0.2116 | 0.1673 | 27.20 |
| 2 | 219029843 | A | C | CXCR1 | rs16858811 | exonic | nsSNP | p.M31R | 0.035420127 | 0.0430 | 0.0260 | 20.62 |
| 2 | 242610172 | A | G | ATG4B | rs138274580 | exonic | nsSNP | p.N330S | 0.00423106 | 0.0242 | 0.0072 | 32.73 |
| 3 | 11400019 | T | C | ATG7 | rs36117895 | exonic | nsSNP | p.V471A | 0.023131095 | 0.0429 | 0.0221 | 34.79 |
| 3 | 49004552 | C | T | ARIH2 | CT_3_48979556 | splicing | . |  | 0.07954616 | 0.0063 | 0.0006 | 17.28 |
| 3 | 49462410 | T | C | NICN1 | rs61729946 | exonic | nsSNP | p.H191R | 0.042140471 | 0.0056 | 0.0159 | 26.01 |
| 3 | 49755481 | G | A | AMIGO3 | GA_3_49730485 | exonic | nsSNP | p.A473V | 0.996375567 | 0.0000 | 0.0053 | 16.97 |
| 3 | 112262957 | T | C | ATG3 | rs3736271 |  | . |  | 0.048997371 | 0.0497 | 0.0370 | 36.28 |
| 3 | 146262699 | C | T | PLSCR1 | CT_3_147745389 | upstream | . |  | 0.103229149 | 0.0078 | 0.0005 | 16.69 |
| 4 | 38799399 | G | T | TLR1 | rs76796448 | exonic | nsSNP | p.H352N | 0.12764363 | 0.0052 | 0.0006 | 11.36 |
| 4 | 74964830 | C | T | CXCL2 | CT_4_75183694 | exonic | nsSNP | p.R3H | 0.046349714 | 0.0066 | 0.0242 | 40.79 |
| 4 | 122085203 | A | G | TNIP3 | rs77437633 | intronic | . |  | 0.005686277 | 0.0338 | 0.0574 | 35.29 |
| 5 | 158743788 | C | A | IL12B | rs3213119 | exonic | nsSNP | p.V298F | 0.011923379 | 0.0191 | 0.0372 | 30.94 |
| 6 | 3285238 | C | G | SLC22A23 | rs79364886 | intronic | . |  | 0.008635698 | 0.0238 | 0.0058 | 40.21 |
| 6 | 32362521 | C | A | BTNL2 | rs28362675 | exonic | nsSNP | p.G454C | 0.017803594 | 0.0252 | 0.0077 | 25.06 |
| 6 | 32363888 | C | T | BTNL2 | rs41441651 | exonic | nsSNP | p.D336N | 0.009701822 | 0.0225 | 0.0056 | 29.43 |
| 6 | 32363893 | G | A | BTNL2 | rs28362679 | exonic | nsSNP | p.S334L | 0.0784402 | 0.0011 | 0.0085 | 24.88 |
| 6 | 32375018 | C | A | BTNL2 | rs28362684 | upstream | . |  | 0.022683304 | 0.0060 | 0.0202 | 27.80 |
| 6 | 36437849 | G | T | KCTD20 | rs41272162 | UTR5 | . |  | 0.044836586 | 0.0201 | 0.0334 | 33.12 |
| 6 | 105609468 | C | T | POPDC3 | rs11961225 | exonic | nsSNP | p.R106Q | 0.006180261 | 0.0415 | 0.0153 | 45.38 |
| 6 | 107077408 | C | T | QRSL1,RTN4IP1 | rs3747791 | upstream | . |  | 0.996414182 | 0.0044 | 0.0000 | 9.36 |
| 6 | 107077507 | C | A | QRSL1 | rs72945092 | UTR5 | . |  | 0.047238468 | 0.0133 | 0.0224 | 33.08 |
| 6 | 137325847 | T | C | IL20RA | rs1555498 | exonic | nsSNP | p.V259I | 0.00201905 | 0.0234 | 0.0034 | 39.67 |
| 6 | 137540536 | G | A | IFNGR1 | rs17181457 | UTR5 | . |  | 0.005877153 | 0.0877 | 0.0289 | 11.30 |
| 7 | 75442723 | G | A | CCL24 | rs11465293 | exonic | nsSNP | p.S31F | 0.043164783 | 0.0264 | 0.0391 | 45.83 |
| 7 | 107643901 | T | G | LAMB1 | rs6965420 | upstream | . |  | 0.000195587 | 0.1229 | 0.0677 | 30.15 |
| 8 | 9590755 | T | G | TNKS | rs7001395 |  | . |  | 0.030425004 | 0.0664 | 0.0367 | 77.40 |
| 8 | 23060155 | C | G | TNFRSF10A | CG_8_23116100 | splicing | . |  | 0.044218882 | 0.0018 | 0.0094 | 25.62 |
| 8 | 59465506 | T | C | SDCBP | TC_8_59628060 | upstream | . |  | 0.091593488 | 0.0018 | 0.0115 | 20.43 |
| 8 | 79717500 | G | T | IL7 | GT_8_79880055 | UTR5 | . |  | 0.150010715 | 0.0023 | 0.0063 | 24.44 |
| 8 | 125528135 | G | A | TATDN1 | rs11542673 | exonic | **sSNP** | p.C66C (NM_001146160); p.C113C (NM_032026) | 0.008962693 | 0.0702 | 0.0380 | 43.27 |
| 8 | 125989460 | A | C | ZNF572 | rs10104558 | exonic | nsSNP | p.K317T | 0.019022316 | 0.0186 | 0.0409 | 37.96 |
| 9 | 21217159 | T | G | IFNA16 | rs41313958 | exonic | nsSNP | p.H49P | 0.195858163 | 0.0025 | 0.0072 | 24.08 |
| 9 | 21409094 | C | T | IFNA8 | rs28383785 | upstream | . |  | 0.006486554 | 0.0297 | 0.0120 | 21.43 |
| 9 | 35612978 | G | A | CD72 | rs34791102 | exonic | nsSNP | p.P234L | 0.048973214 | 0.0720 | 0.0441 | 27.03 |
| 9 | 115652255 | G | C | SLC46A2 | rs76734829 | exonic | nsSNP | p.P236R | 0.204119612 | 0.0029 | 0.0068 | 25.03 |
| 10 | 5541277 | T | C | CALML5 | TC_10_5531277 | exonic | nsSNP | p.K42R | 0.103104431 | 0.0023 | 0.0082 | 23.38 |
| 10 | 120828969 | G | A | EIF3A | rs7908387 | exonic | sSNP | p.D313D | 0.000155585 | 0.0557 | 0.0217 | 57.066 |
| 11 | 19722051 | G | A | MRPL23 | rs2240197 | exonic | nsSNP | p.G32S | 0.036381281 | 0.0323 | 0.0145 | 52.84 |
| 12 | 66603980 | A | G | IRAK3 | rs56001649 | exonic | **sSNP** | p.K84K (NM_001142523); p.K145K (NM_007199) | 0.029071272 | 0.0038 | 0.0146 | 26.28 |
| 12 | 69254076 | C | T | CD4 | rs28919570 | exonic | nsSNP | p.R86W (NM_001195014); p.R265W(NM_000616) | 0.03729543 | 0.0102 | 0.0019 | 26.02 |
| 12 | 76494847 | C | T | CD163 | rs4883263 | exonic | nsSNP | p.I342V | 0.043800118 | 0.0534 | 0.0283 | 48.51 |
| 12 | 129373247 | C | T | GLT1D1 | rs73440307 | exonic | nsSNP | p.A94V | 0.040126261 | 0.0120 | 0.0013 | 26.09 |
| 12 | 132396679 | G | A | ULK1 | rs12317065 | intronic | . |  | 0.005923946 | 0.0130 | 0.0450 | 84.40 |
| 15 | 28443658 | G | T | HERC2 | rs8041209 | splicing | . |  | 2.75E-05 | 0.0915 | 0.0382 | 51.54 |
| 15 | 28502279 | G | A | HERC2 | rs11631797 | exonic | sSNP | p.G815G | 4.27E-07 | 0.2332 | 0.1419 | 47.32 |
| 16 | 3820667 | C | T | CREBBP | rs3025694 | exonic | **sSNP** | p.P928P (NM_004380 ); p.P890P (NM_001079846) | 0.034055738 | 0.0081 | 0.0010 | 21.53 |
| 16 | 8989042 | T | A | USP7 | rs79003022 | intronic | . |  | 0.003910139 | 0.0141 | 0.0320 | 39.17 |
| 16 | 13069231 | C | T | TPSD1 | CT_16_1246924 | exonic | nsSNP | p.A127V | 0.158085495 | 0.0012 | 0.0103 | 13.60 |
| 16 | 29675855 | T | C | SPN | rs34710374 | exonic | nsSNP | p.V269A | 0.047595128 | 0.0132 | 0.0268 | 37.29 |
| 16 | 85932351 | C | T | IRF8 | rs188602 | upstream | . |  | 0.00076995 | 0.0864 | 0.0431 | 45.93 |
| 17 | 34328461 | G | A | CCL15 | rs854625 | exonic;splicing | nsSNP | :p.I24T | 0.027758893 | 0.0733 | 0.0402 | 51.34 |
| 17 | 45811354 | C | G | TBX21 | rs41444548 |  | . |  | 0.007823996 | 0.0531 | 0.0795 | 75.29 |
| 17 | 73401941 | C | G | GRB2,MIR3678 | rs55984901 |  | . |  | 0.03783531 | 0.0608 | 0.0393 | 82.08 |
| 19 | 8118000 | A | G | CCL25 | rs960173 | exonic | nsSNP | p.T23A | 0.004616646 | 0.0153 | 0.0366 | 51.34 |
| 19 | 33482821 | G | A | RHPN2 | rs79314177 | exonic | nsSNP | p.R518C | 0.016514271 | 0.0484 | 0.0257 | 58.35 |
| 19 | 33517515 | C | T | RHPN2 | rs28626308 | exonic | nsSNP | p.R70Q | 0.031867713 | 0.0624 | 0.0417 | 37.55 |
| 19 | 38939408 | C | T | RYR1 | rs10406027 | exonic | **sSNP** | p.A359A | 0.029732802 | 0.1194 | 0.1512 | 51.43 |
| 19 | 38990336 | C | T | RYR1 | rs2228071 | exonic | **sSNP** | p.C2363C (NM_001042723); p.C2363C (NM_000540 ) | 0.049269004 | 0.0293 | 0.0492 | 43.46 |
| 20 | 43034783 | C | T | HNF4A | rs736823 | exonic | **sSNP** | p.A67A (NM_178850 ); p.A45A (NM_001030004); p.A67A (NM_178849, NM_000457); p.A45A (NM_175914); p.A45A ( NM_001030003) | 0.026328085 | 0.0745 | 0.0480 | 61.69 |
| 20 | 62325690 | T | C | RTEL1-TNFRSF6B | rs2738786 | ncRNA | . |  | 0.005311965 | 0.0173 | 0.0410 | 42.97 |
| 21 | 34799244 | A | C | IFNGR2 | AC_21_33721114 | exonic | nsSNP | p.I156L | 0.222639895 | 0.0017 | 0.0047 | 19.23 |
| 22 | 37333406 | T | G | CSF2RB | rs16997510 | intronic | . |  | 0.013537519 | 0.0321 | 0.0120 | 31.40 |
