# Supplementary material for: Pooled Sequencing of 531 Genes in Inflammatory Bowel Disease Identifies an Associated Rare Variant in BTNL2 and Implicates Other Immune Related Genes
Source: PLoS Genet. 2015 Feb 11;11(2):e1004955. doi: 10.1371/journal.pgen.1004955 (PMC4335459; doi:10.1371/journal.pgen.1004955)
Supplement: S6 Table — (DOCX) [file pgen.1004955.s011.docx]

Table S6

|  |  |  |  |  |  | **IBD p val assoc with conditioning marker/s** | | | |
| --- | --- | --- | --- | --- | --- | --- | --- | --- | --- |
| **Variant description** | **dbSNP ID** | **risk allele** | **Ca-Freq** | **Co-Freq** | **p value assoc** | **rs28362675** | **r9264942** | **rs477515** | **r9264942 & rs477515** |
| *BTNL2* p.G454C | rs28362675 (A>C) | **A** | 0.0082 | 0.0024 | 0.00225 | 1 | 4.83E-05 | 0.0045 | 0.000556 |
| *Common* ***CD*** *GWAS SNP* | r9264942 (G>A) | **G** | 0.3694 | 0.3492 | 0.11358 | 0.00147 | 1 | 0.047 |  |
| *Common* ***UC*** *GWAS SNP* | rs477515 (G>A) | **G** | 0.6828 | 0.6468 | 4.09E-03 | 7.76E-03 | 2.66E-03 | 1 |  |
