# Supplementary material for: Pooled Sequencing of 531 Genes in Inflammatory Bowel Disease Identifies an Associated Rare Variant in BTNL2 and Implicates Other Immune Related Genes
Source: PLoS Genet. 2015 Feb 11;11(2):e1004955. doi: 10.1371/journal.pgen.1004955 (PMC4335459; doi:10.1371/journal.pgen.1004955)
Supplement: S7 Table — (DOCX) [file pgen.1004955.s012.docx]

Table S7

| Haplotype | |  |  |  |  |  |  |  |  |
| --- | --- | --- | --- | --- | --- | --- | --- | --- | --- |
| rs477515 | p.G454C | Case | Control | Ca-Freq | Co-Freq | Odds-R | 95%Lo | 95%Hi | P |
| A | A | 3.103 | 1.932 | 0.001767 | 0.000775 | 2.854 | 0.165 | 49.38 | 0.585598 |
| **G** | A | 14.9 | 4.068 | 0.008484 | 0.001632 | 6.509 | 1.865 | 22.72 | 0.001316 |
| A | C | 492.9 | 876.1 | 0.2807 | 0.3516 | 1 | 1 | 1 | 1 |
| **G** | C | 1245 | 1610 | 0.7091 | 0.646 | 1.375 | 1.202 | 1.572 | 2.92E-06 |
